# Comparative Evaluation of Pain Scores during Periodontal Probing with or without Anesthetic Gels

**DOI:** 10.1155/2016/5768482

**Published:** 2016-02-29

**Authors:** Ashank Mishra, Mandapathi Priyanka, Koppolu Pradeep, Krishnajaneya Reddy Pathakota

**Affiliations:** ^1^Department of Periodontics, Sri Sai College of Dental Surgery, Vikarabad, Telangana 501101, India; ^2^Department of Preventive Dental Sciences, AlFarabi Colleges, Riyadh 11691, Saudi Arabia

## Abstract

*Context*. The initial periodontal examination which includes full-mouth periodontal probing is one of the discomforting procedures for a patient.* Aim.* To evaluate the efficacy of two local anesthetic gels in the reduction of pain during periodontal probing using Florida probe in CGP patients in comparison with manual probing.* Materials and Methods*. Ninety systemically healthy patients with moderate to severe CGP patients were recruited. In each patient, the quadrants were randomly assigned to manual probing with UNC-15 probe, probing with Florida probe, and Florida probing with lidocaine 10% gel and with benzocaine 20% gel. In the quadrants undergoing probing with anesthetic gels, the sites were isolated and the gel was injected using syringe and a blunt-end cannula. Pain was measured using 10 mm horizontal VAS.* Statistical Analysis*. The analysis was carried out using SPSS version 18. The comparison of mean VAS scores was done using repeated measures ANOVA with post hoc Bonferroni test.* Results*. Mean VAS for manual probing was significantly more than Florida probing. Further, the mean VAS score for Florida probing was higher than the two gels.* Conclusion*. It is suggested that the gels might be useful in reducing pain experienced during full-mouth periodontal probing in patients with CGP.

## 1. Introduction

Periodontal probing is one of the basic baseline clinical examination procedures which is carried out to assess the disease severity in a periodontitis patient. Measurement of clinical parameters like probing depth, clinical attachment level (CAL) gives us a basic idea regarding the evaluation of response to periodontal therapy. The purpose of full-mouth periodontal examination involves periodontal probing at six sites (mid-buccal, distobuccal, mesiobuccal, mesiolingual, midlingual, and distolingual) per tooth on all teeth.

In untreated cases, full-mouth periodontal probing has always been an extremely painful experience which is mainly due to the persisting inflammation of the periodontal tissues [1]. The pain experienced during this baseline examination procedure has always been a matter of concern but is not taken care of. In an untreated periodontal site, probing leads to penetration of the periodontal probe into the surrounding connective tissue, which is heavily infiltrated with chronic inflammatory cells, whereas in a treated, noninflamed site, the periodontal probe does not penetrate through the epithelium at the base of the pocket which was proved histologically in earlier studies [2].

Undoubtedly, there are no precise clinical approaches to reduce this pain. Injection type of anesthesia is a well known method, but it has its own limitations which makes it difficult to implement. Topical anesthetics in the form of creams, ointments, jellies, or sprays were also tried in the past but they are not so effective as they have superficial penetration and shorter duration of action [3–5].

The most common method used in epidemiological studies to assess pain is VAS (Visual Analogue Scale). It is simple, reliable, and most accepted method [6, 7]. It was demonstrated in a study that the clinical signs of inflammation like bleeding on probing and so forth, before and after periodontal therapy, were related to the VAS scores obtained. It was also noted that the periodontal inflammation directly influenced the intensity of pain and discomfort associated with periodontal probing.

Recently, the efficacy of EMLA (25 mg/g lidocaine and 25 mg/g prilocaine) has been evaluated as an intrapocket anesthetic gel. At room temperature, it is a low-viscosity fluid, exhibiting a property of transforming into an elastic gel when applied into the periodontal pocket. It was concluded that the gel may be used for full-mouth periodontal probing in untreated periodontitis patients who find it difficult during the procedure. This gel was successfully used to reduce pain during SRP (scaling and root planning) as reported in earlier studies [8–11].

The aim of the present study was to evaluate the efficacy of two anesthetic gels (lidocaine 10% and benzocaine 20%) in the reduction of pain during periodontal probing using Florida probe in untreated patients with chronic generalized periodontitis (CGP) in comparison with manual probing and probing using Florida probe.

## 2. Materials and Methods

The study was carried out as a randomized, split-mouth clinical trial comparing the efficacy of local delivery of anesthetic gels, lidocaine 8% and benzocaine 20% using Florida probing in comparison with manual and Florida probing to reduce pain in untreated periodontal patients. A total of 90 patients were included. Ethical clearance was obtained from the Institutional Ethics Committee.

### 2.1. Study Population

90 untreated CGP patients (50 males and 40 females, aged 29 years to 53 years; mean age: 39.60 ± 7.32 years) were recruited from the Department of Periodontics, at the baseline, case history was recorded, and other clinical parameters were measured. The subjects were selected according to the following inclusion criteria: (1) 28 to 60 years of age; (2) presence of ≥1 tooth with PD ≥ 5 mm in each quadrant; (3) patients who needed to have a minimum of two incisors, one canine, one premolar, and one molar in all the quadrants; (4) patients who should not have undergone any sort of periodontal therapy in the past 12 months. The following individuals were excluded from the study: (1) patients requiring prophylactic antibiotics before periodontal probing, (2) patients suffering from any mental disorders or with any chronic pain problems, (3) patients suffering with coagulation/bleeding disorders or on anticoagulants, (4) pregnant or lactating women, (5) patients with congenital or idiopathic methemoglobinemia, (6) patients reporting hypersensitivity to lidocaine/benzocaine, (7) patients taking nonsteroidal anti-inflammatory drugs in the 3 days before participation in the study, and (8) patients having acute periodontal pain, pulpitis, abscesses, or other acute infections.

**Figure 1:**
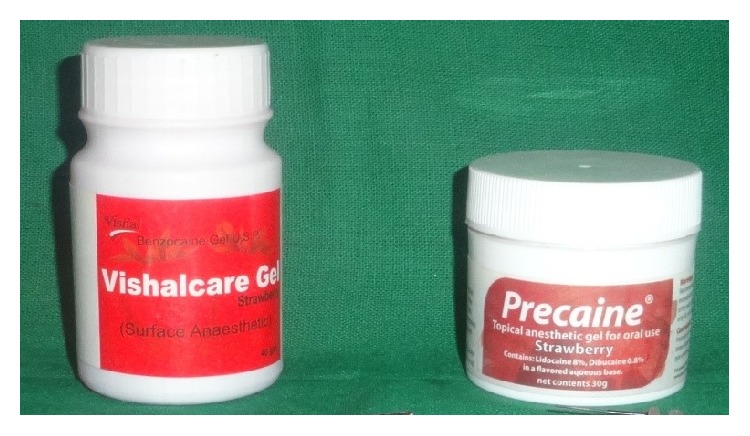
Gels used. Benzocaine 20% (left side) and lidocaine 8%.

The study was performed in a split-mouth manner, incorporating all the four quadrants. Computer generated block randomization was carried out in recruiting the patients. The quadrants receiving either manual or Florida probing were carried out initially for two reasons: to reduce any effect of the incorporation of gels in the other quadrants and to obtain a definitive score for probing without the gels. The gels were colour and consistency matched to eliminate bias. Both were strawberry flavoured (Figure 1). Gels were filled in 2 mL graduated syringes with a blunt-ended needle cannula.

**Figure 2:**
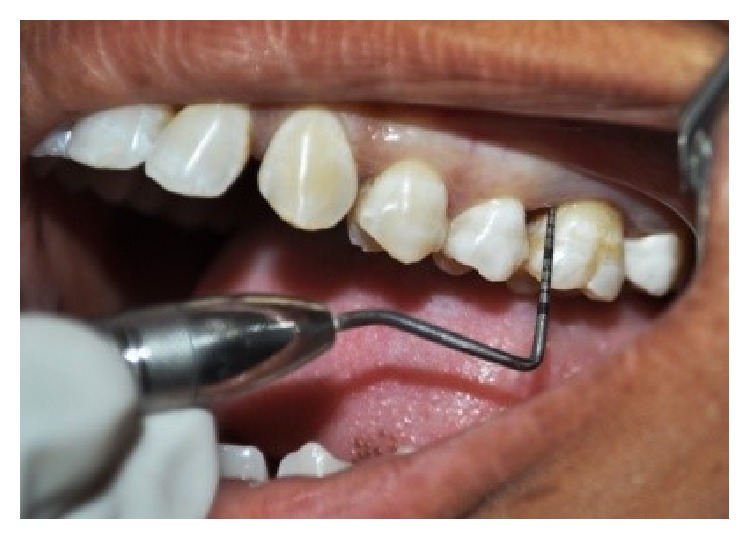
Manual probing alone in one randomized quadrant.

**Figure 3:**
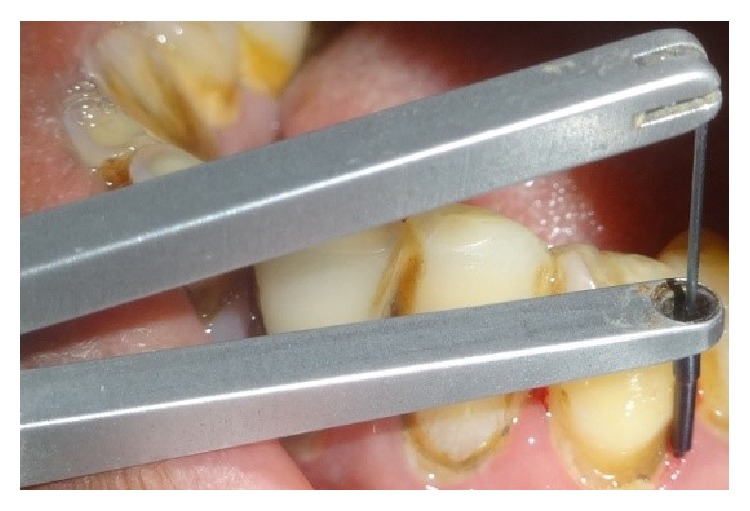
Florida probing alone in a randomized quadrant.

**Figure 4:**
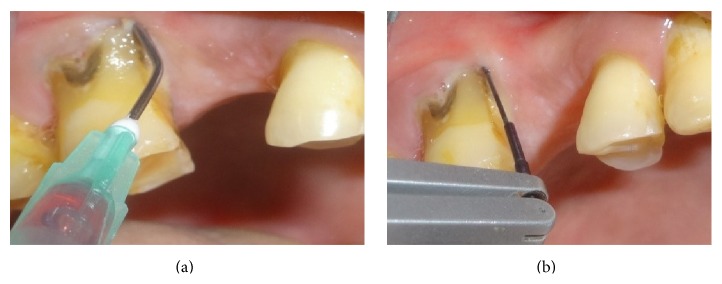
(a) Administration of 20% benzocaine gel. (b) Administration of 20% benzocaine gel, followed by Florida probing.

**Figure 5:**
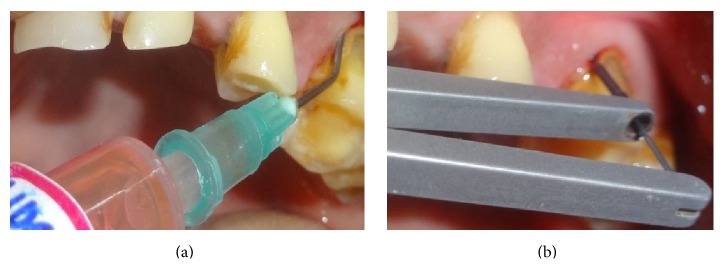
(a) Administration of 10% lidocaine gel. (b) Administration of 10% lidocaine gel followed by Florida probing.

In quadrants receiving manual probing, a periodontal probe (Hu Freidy UNC-15 probe) was used at six sites per tooth (Figure 2). After completion of probing in the quadrant, the patient was asked to fill the pain assessment for probing in the respective quadrant. Florida probing alone was carried out in a randomized quadrant without prior application of either of the gels (Figure 3). For the quadrants receiving the gels, the area was dried and thoroughly isolated with cotton rolls. The gels were administered around the gingival margins of the teeth and also into the periodontal pockets. Central incisors were excluded from the application of gels in order to avoid cross side contamination with either of these gels. The gels were left in situ for a period of 30 seconds after application and before Florida probing began (Figures 4(a), 4(b), 5(a), and 5(b)). After washing the quadrants with water spray for 30 seconds, the patient was then asked to fill the VAS score for probing in the respective quadrant using a 10 mm VAS scale with the left endpoint marked as “no pain” and the right endpoint marked as “worst imaginable pain.” Patients were asked to wait for 30 minutes before the other gel was applied. By obtaining the results in this manner, each patient was effectively acting as their own control, producing four separate VAS scores for probing in each of the quadrants probed.

### 2.2. Statistical Analysis

All the analysis was done using SPSS version 18. A *p* value of <0.05 was considered as statistically significant. Comparison of mean VAS scores was done using repeated measures ANOVA with post hoc Bonferroni test.

Repeated measures ANOVA with post hoc Bonferroni test revealed overall significant difference in the mean VAS score among the 4 types of probing (*p* < 0.001). Further, manual probing had significantly higher mean VAS score than Florida probing, lidocaine and benzocaine groups. Similarly, Florida probing also had significantly higher mean VAS score than lidocaine and benzocaine groups. No other significant differences were found among the groups.

## 3. Results

**Figure 6:**
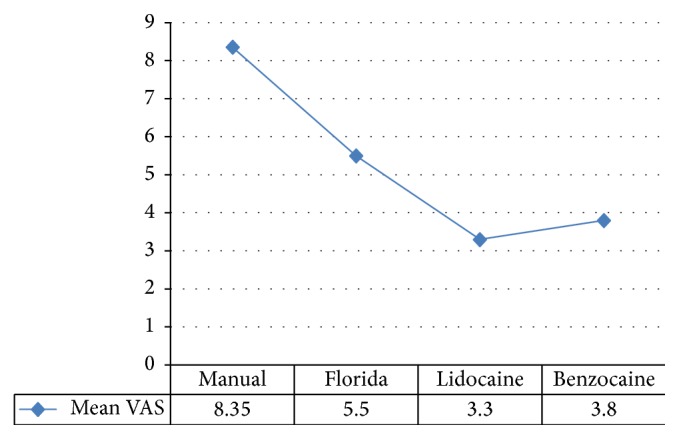
Graphical representation of mean VAS scores.

A total of 90 eligible individuals were selected with mean age of 39.60 ± 7.32 years (Table 1). All 90 patients who participated in the study completed the full-mouth probing examinations as mentioned above, with no adverse events being reported. The mean ± SD VAS scores were 8.35 ± 1.18 mm for manual probing, 5.50 ± 1.76 mm for Florida probing group, 3.30 ± 1.56 mm for lidocaine group, and 3.80 ± 1.51 mm for benzocaine group (Table 2, Figure 6). Repeated measures ANOVA with post hoc Bonferroni test revealed that the mean VAS for manual probing was significantly more than Florida probing. Further, the mean VAS score for Florida probing was higher than the two gels. There was no statistically significant difference in mean VAS scores between the lidocaine and benzocaine groups.

## 4. Discussion

In the present study Florida probe was used, as it facilitates delivery of equal amount of force (15 g) during periodontal probing and, thus, this probe can be used for standardization of force especially while comparing efficacy of two different methods of probing [12].

Florida probe is a third-generation periodontal probe which combines controlled force application, automated measurement, and computerized data capture. Jeffcoat et al. (1986) [13] noted that each of the 3rd-generation probes offers improved measurements resolution over 1st- and 2nd-generation probes. This improved resolution leads to a more continuous distribution of measurements and eliminates problems involved in rounding to whole integer values. The Florida probe measures resolution of 0.1 mm. An important advantage of these probes is automated data capture that facilitates data entry into patient records and eliminates errors in data transcription, which may occur with other probing methods.

The calibration of pressure before periodontal probing facilitates delivery of equal amount of force during periodontal probing. Thus, this probe can be used for standardization of force especially while comparing efficacy of two different methods of probing. In between the applications of the anesthetic gels in two different quadrants, 30-minute interval was given to wash out the anesthetic effect of the firstly applied gel.

The randomization was done for allotting the quadrant to the type of probing to be done.

Winning et al. (2012) evaluated the efficacy of Oraqix in the reduction of pain during periodontal probing and concluded that the gel can be used for full-mouth periodontal probing. It was a randomized controlled trial, but the main drawback of the study was the lack of standardization of the force [14].

Previously in the studies which used the gels in the SRP procedures, it was found that the gel (Oraqix) can be used in reducing pain scores. But the studies were basically of parallel group design [1, 10, 11]. Unlike these studies, the present study increased the efficiency in statistical testing by using split-mouth design.

As reported in earlier studies, the pain perceived during SRP is basically due to two sources: one is due to manipulation of the surrounding gingival tissues, and the second is due to disturbance of the dentinal tubules, which elicits pain from the nonanesthetized nociceptive fibers present in the dental pulp. The anesthetic gels are not known to provide any form of pulpal anesthesia; hence in procedures like periodontal probing, in which the pain is mainly from manipulation of periodontal tissues, the anesthetic gels may be more effective when compared to SRP procedures.

## 5. Conclusion

The use of local anesthetic gels in baseline periodontal probing provides a significant reduction in pain in untreated periodontitis patients. It suggests that the gels may be useful for those patients who find the full-mouth periodontal probing experience particularly painful when compared to methods which have been tried earlier. Additional studies with larger sample sizes should be conducted to compare the efficacy between the two gels used. In addition for full-mouth periodontal probing, it is significant to know the level of anesthesia achieved and the accuracy of the periodontal probing.

## Figures and Tables

**Table 1:** Demographic data.

		*N*	%	Age (mean ± SD)
Sex	Male	50	54.0%	39.60 ± 7.32
	Female	40	46.0%	

**Table 2:** Intergroup comparison of VAS scores.

	Mean ± SD	*p* value	Post hoc test
Manual	8.35 ± 1.18	<0.001; sig	Manual > Florida, lidocaine, benzocaine Florida > lidocaine, benzocaine
Florida	5.50 ± 1.76		
Lidocaine	3.30 ± 1.56		
Benzocaine	3.80 ± 1.51		
